# Weighted gene co-expression network analysis reveals immune evasion related genes in *Echinococcus granulosus* sensu stricto

**DOI:** 10.3389/ebm.2024.10126

**Published:** 2024-02-29

**Authors:** Ismael Pereira, Gabriela Prado Paludo, Christian Hidalgo, Caroll Stoore, María Soledad Baquedano, Carolina Cabezas, Martín Cancela, Henrique Bunselmeyer Ferreira, Macarena Bastías, Aníbal Riveros, Claudio Meneses, Leonardo Sáenz, Rodolfo Paredes

**Affiliations:** ^1^ Laboratorio de Medicina Veterinaria, Escuela de Medicina Veterinaria, Facultad de Ciencias de la Vida, Universidad Andres Bello, Santiago, Chile; ^2^ Programa de Doctorado en Ciencias Silvoagropecuarias y Veterinarias, Universidad de Chile, Santiago, Chile; ^3^ Laboratório de Genômica Estrutural e Funcional, Centro de Biotecnologia, Universidade Federal do Rio Grande do Sul (UFRGS), Porto Alegre, RS, Brazi; ^4^ Núcleo de Investigaciones Aplicadas en Ciencias Veterinarias y Agronómicas, Facultad de Medicina Veterinaria y Agronomía, Universidad de Las Américas, Sede Santiago Centro, Santiago, Chile; ^5^ Centro de Biotecnología Vegetal, Facultad de Ciencias de la Vida, Universidad Andrés Bello, Santiago, Chile; ^6^ Laboratorio de Vacunas Veterinarias, Facultad de Ciencias Veterinarias y Pecuarias, Universidad de Chile, Santiago, Chile

**Keywords:** cystic echinococcosis, *Echinococcus granulosus*, RNAseq, WGCNA, co-expression network

## Abstract

Cystic echinococcosis (CE) is a zoonotic disease caused by the tapeworm *Echinococcus granulosus* sensu lato (s.l). In the intermediate host, this disease is characterized by the growth of cysts in viscera such as liver and lungs, inside of which the parasite develops to the next infective stage known as protoscoleces. There are records that the infected viscera affect the development and morphology of *E. granulosus* s.l. protoscolex in hosts such as buffalo or humans. However, the molecular mechanisms that drive these differences remains unknown. Weighted gene co-expression network analysis (WGCNA) using a set of RNAseq data obtained from *E. granulosus* sensu stricto (s.s.) protoscoleces found in liver and lung cysts reveals 34 modules in protoscoleces of liver origin, of which 12 have differential co-expression from protoscoleces of lung origin. Three of these twelve modules contain hub genes related to immune evasion: tegument antigen, tegumental protein, ubiquitin hydrolase isozyme L3, COP9 signalosome complex subunit 3, tetraspanin CD9 antigen, and the methyl-CpG-binding protein Mbd2. Also, two of the twelve modules contain only hypothetical proteins with unknown orthology, which means that there are a group of unknown function proteins co-expressed inside the protoscolex of liver CE cyst origin. This is the first evidence of gene expression differences in protoscoleces from CE cysts found in different viscera, with co-expression networks that are exclusive to protoscoleces from liver CE cyst samples. This should be considered in the control strategies of CE, as intermediate hosts can harbor CE cysts in liver, lungs, or both organs simultaneously.

## Impact statement

This is the first report of a Weighted gene co-expression network analysis in *Echinococcus granulosus* sensu stricto protoscolex stage. These networks are useful in understanding parasite basic biology and provide the baseline for further research. Immune evasion is a relevant topic in the host-parasite interaction, and the identified networks could provide new molecular targets in the medical treatment of this disease, such as the COP9 signalosome subunit.

## Introduction

Cystic Echinococcosis (CE) is a zoonotic larval disease, caused by the infection of the tapeworm *Echinococcus granulosus* sensu lato (s.l.), and affects both animals and humans globally. According to the World Health Organization (WHO), the global burden of trying to stop this zoonosis exceeds three billion US dollars every year [1]. CE is characterized by the development of cysts (formerly called hydatid cysts) in the viscera (mainly, liver and lungs) of intermediate hosts such as cattle and sheep, among other herbivores, whereas humans act as dead-end hosts [2]. CE cysts are comprised of three layers: the germinal and laminated layer of parasite origin, and an adventitial layer, which is the result of the host immune response against the parasite [3]. The germinal layer produces protoscoleces, the infective stage for the definitive host (dogs and other canids), who become infected when consuming viable protoscoleces [4]. *E. granulosus* s.l. is a cluster that groups different species of *Echinococcus* such as *E. equinus*, *E. ortleppi*, *E. canadensis*, *E. felidis* and *E. granulosus* sensu stricto (s.s.) [5]. The latter, which corresponds to the sheep-dog cycle, is the main *E. granulosus* s.l. species that infects humans [6], and it is found in Asia, Europe, Oceania and the Americas [7]. For causes that remain to be fully understood, some *E. granulosus* s.l. CE cysts are unable to produce viable protoscoleces and are termed non-fertile CE cysts [3]. Since cattle and sheep host can harbor simultaneously fertile and non-fertile CE cysts in the same viscera, from the same *E. granulosus* s.s. species [2], there must be microenvironment factors that affect parasite development and shape the host immune response.

Differences in morphology and fertility of CE cysts regarding infected organ have been previously described. In *E. granulosus* s.l. cysts from buffalo, the maturation rate of protoscoleces of liver origin was found to be different as compared to those of lung origin [8]. Also, a morphological study showed that larger protoscoleces were found in lungs CE cysts compared to those found in liver CE cysts, and presented size variability depending on the organ localization, among other variables [9]. So far, the identification of the molecular mechanisms that drive these differences remain unknown.

In a “classic approach” of gene expression profiles, the analyses focus on the individual genes, ignoring completely the interactions and expression minor variation among them, leaving a gap of information as genes play roles not by isolation but by interaction with each other. The gene-to-gene co-expression analysis emerged as an approach to solve the gene interaction problems [10]. Weighted gene co-expression network analysis (WGCNA) has been applied to many studies since late 2008, when the R package WGCNA was released [11], and then growing exponentially, being the 2012 the first year to reach 10 articles per year with this methodology and in the 2021 reaching more than 600 articles per year already published. Focusing on high complexity and multifactorial biological problems, WGCNA can be used as a data exploratory tool or as a gene screening method; having applications in gene expression and protein interaction data, among others [12]. Comparing gene expression networks (not only an individual gene) between set samples may identify modules of co-expressed genes and expression profiles of intramodular hub genes inside an interesting module, showing the Connectivity (the relative importance of a gene in a network). This approach may be performed to identifying this hub genes as possible therapeutic targets based on the constructed network [13, 14].

WGCNA has been already proposed to explore the parasite—host interaction. In human patients with chronic Chagas disease (*Trypanosoma cruzi*), hub genes were found associated with immune cell signaling pathways, T cell activation and B cell cellular immunity, although gene expression analysis of the parasite were not done [15]. In another instance, transcriptomic analyses of the freshwater snail *Oncomelania hupensis*, were performed after 3 different times of invasion with the parasite *Schistosoma japonicum*, finding a module related to ribosomes, translation, mRNA processing, among others, associated with *Schistosoma* infection [16]. Another work explored the presence of non-conserved modules and its hub genes between complicated and uncomplicated disease generated by protozoan *Plasmodium falciparum*, finding differences among those groups and identifying key genes for the development of the parasite [17]. Similar experiences have been performed in *Trypanosoma brucei* species, being those findings key in the potential identification of molecular targets to control the disease [18]. These findings show that WGCNA is a useful tool in the analysis of transcriptomic data in samples from parasite diseases, although most of them are focused on the host instead of the parasite.

Thus, it would not be unexpected that more complex statistical methods, such as the generation of co-expression networks, could help to elucidate subtle differences associated with parasite survival in different host organs. Therefore, to understand the differences in gene expression between *E. granulosus* s.s. protoscoleces found in liver and lung CE cysts, a WGCNA was performed. We found several modules with hub genes coding for hypothetical protein products and immune evasion related modules, revealing gene co-expression networks. Furthermore, we discuss possible molecular mechanisms performed by *Echinococcus granulosus* s.s. in response to adverse environments found in different organs that they can parasitize.

## Materials and methods

### Sample collection and protoscolex viability

Fresh CE cysts were obtained from sheep liver and lung in a slaughterhouse. All CE cysts were visually inspected and each one was considered an individual sample. The protoscoleces obtained were washed with PBS pH 7.2. The viability of protoscoleces was assessed with the trypan blue exclusion test. All samples showing less than 90% of viability were discarded. Suitable protoscolex samples (>90% viability) were conserved in RNAlater^®^ solution and frozen at −80°C.

### DNA isolation and genotype identification

To corroborate the genetic identity and molecular diversity of the protoscolex samples, DNA was extracted and the full length of the mitochondrial cox1 was amplified and sequenced, as previously described [2]. Briefly, DNA isolation was performed with WIZARD Plus SV Genomic Purification Systems kit (PROMEGA). The *E. granulosus* s.s. mithocondrial haplotypes were identified using the full length of the cytochrome C oxidase subunit gene (cox1, 1609bp). The PCR was performed with 30–100 ng of DNA, using 0.5 U DNA Taq Pol, 1X Buffer Taq DNA Pol (20 mM Tris-HCl, pH 8.4, 50 mM KCl), 0.04 mM mix dNTP, 1.5 mM MgCl_2_ and 20 pmol of each primer (5′-TTA CTG CTA ATA ATT TTG TGT CAT-3′ forward and 5′-GCA TGA TGC AAA AGG CAA ATA AAC-3′ reverse) in a final volume of 25 µL. After the amplification, the amplicons were purified and sequenced. Only *E. granulosus* s.s. samples were used in further experiments.

### RNA isolation and cDNA library preparation

Total RNA was extracted from CE cyst protoscolex with Rneasy^®^ mini kit, then it was measured by fluorometry (Qubit 2.0, Invitrogen). The inclusion criteria were: high purity (A260/A280 > 1.8), 1ug of total RNA, and an electropherogram similar to the reported from [19]. Libraries were made with Illumina TruSeq^®^ Stranded mRNA Library Prep Kit, following the manufacturer’s protocol. These libraries were sequenced in paired-end on Illumina HiSeq4000 platform.

### WCGNA analysis

Twelve protoscolex samples were obtained; six samples from sheep liver CE cysts and six samples from sheep lung CE cysts. Six of these samples, three liver and three lung CE cyst samples, where obtained from the same sheep. RAW files were adapter trimmed with TrimmGalore to remove low quality sequences, primers and adapters and trimmed to a set length cutoff of 50 bp. Quality control was performed with FastQC, the alignment was made with STAR and the reference genome was GCA_000524195.1 ASM52419v1. Data normalization and gene count estimation were performed by DESeq2 as described elsewhere [20]. The Gene co-expression calculation was performed with WCGNA R package with bi-weight mid-correlation method [11, 21]. We used a soft-thresholding power for the network construction to obtain the closest scale-free topology. In that regard, we used the β power value of 30 with SFT.R.sq = 0.835000.

Gene clusters were identified by hierarchical method and the expression values were summarized into module eigengenes (ME). Calculation of Intramodular Hub Genes (kME) was performed correlating the expression of each gene and its ME. To choose the hub genes, we picked the 10% of the top genes from the module that also showed kME values higher than 0.95. The set of genes associated with each cluster were categorized and functionally enriched based gene ontology (GO) annotations from the genome (available in WormBase ParaSite: PRJNA182977) using the Blast2GO software. WGCNA co-expression network and only connections with a value > 0.1 were selected to generate the Gen-Gen Interaction network using Cytoscape. The homology search was performed through the web version of BLAST [22].

## Results

### Functional analysis of co-expressed genes from liver

**FIGURE 1:**
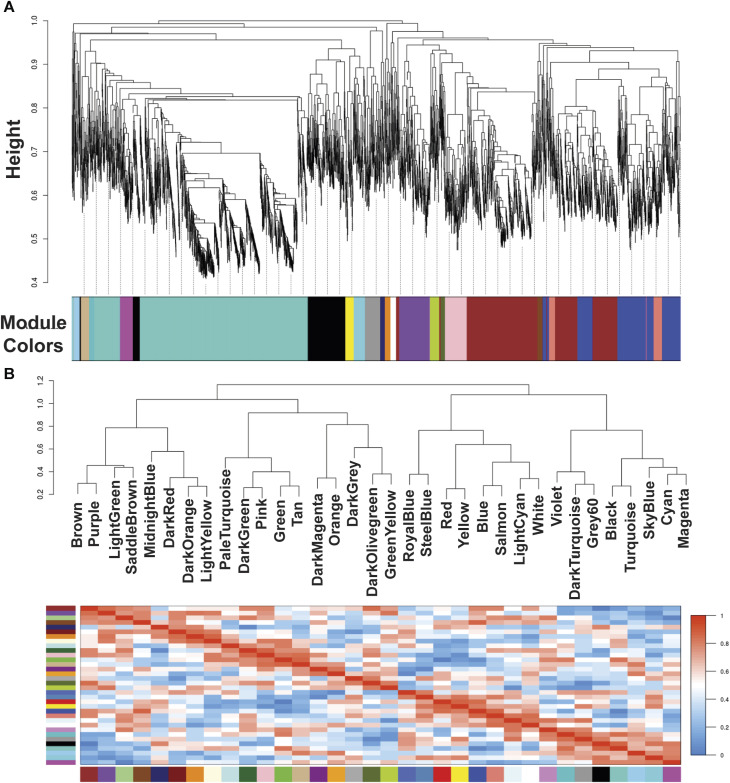
Module colors identification and Cluster dendrogram. **(A)** Identification of gene co-expression modules in *Echinococcus granulosus* sensu stricto protoscoleces from sheep liver CE cyst samples. The y-axis (Height) denotes the co-expression distance, and the x-axis corresponds to genes. Each color in the horizontal bar, but grey, represents a module. **(B)** Clustering dendrograms of consensus module eigengenes for identifying meta-modules and Heatmap of eigengene adjacencies in the consensus eigengene network. Each row and column correspond to one eigengene (labeled by consensus module color). Within the heatmap, red indicates high adjacency (positive correlation) and green low adjacency (negative correlation) as shown by the color legend. CE, cystic echinococcosis.

**FIGURE 2:**
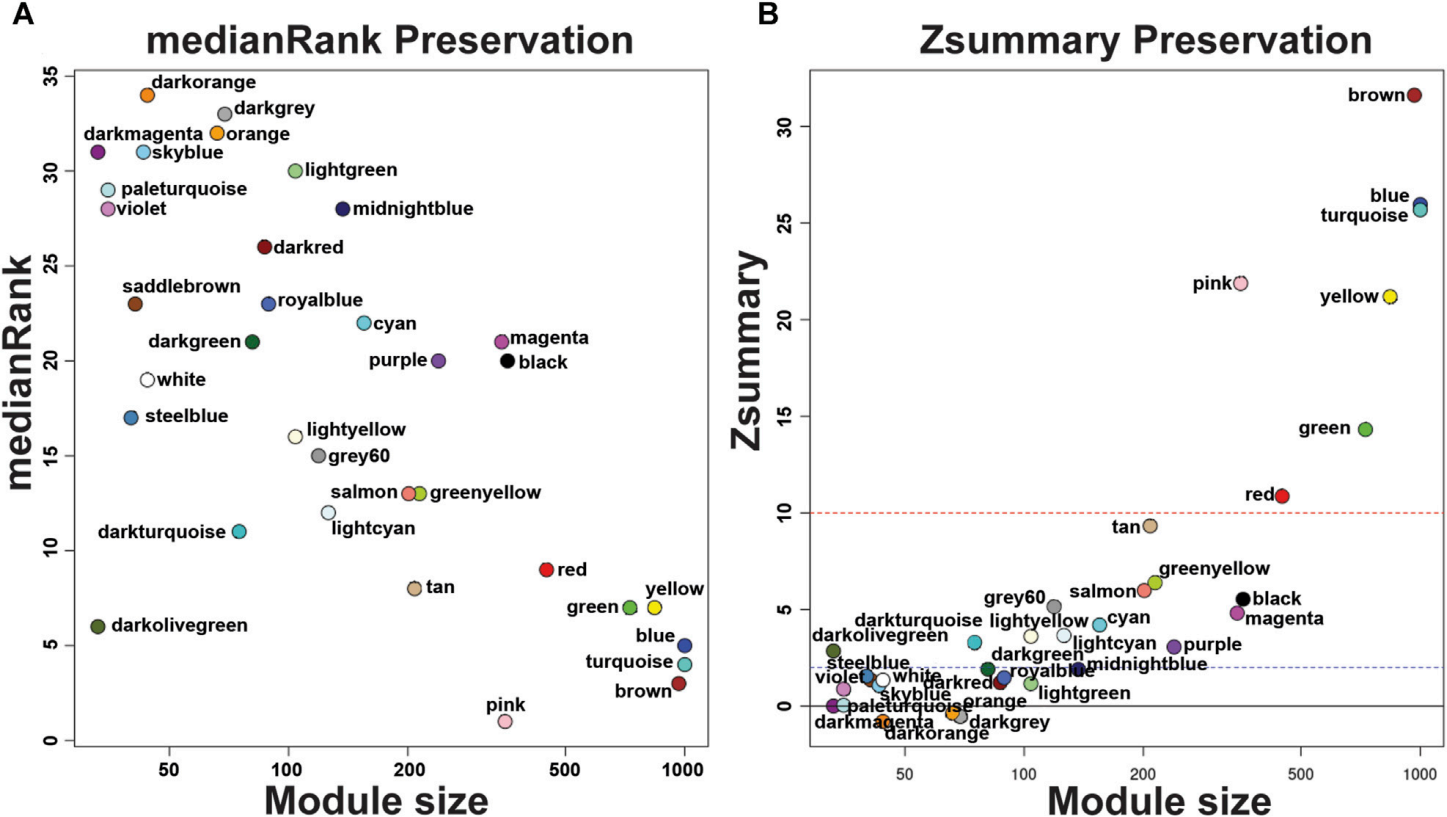
medianRank Preservation graph and Preservation Zsummary graph of *Echinococcus granulosus* sensu stricto protoscoleces genes from sheep liver CE cysts. **(A)** medianRank Preservation graph. Modules close to zero indicates a high degree of module preservation. **(B)** Preservation Zsummary graph. The dashed blue line indicates threshold Z = 2 and the red dashed line indicates threshold Z = 10. Each dot and its color belong to a module. Modules over the red line (high threshold) show strong evidence of conservation. Modules under the blue line (low threshold) show non-conservated modules. CE, cystic echinococcosis.

Our group previously described that the transcriptome analysis of protoscoleces shows a difference in immune modulation gene expression from cattle compared with sheep CE cysts. Besides, RNA-seq data were used to generate specific gene co-expression networks for each organ, lung and liver and determine the differentially immune genes expressed [23]. Using the information of the transcriptome analysis indicated above, through the WGCNA package pipeline, we identified that none of the transcriptome samples were identified as outlier and all 12 (6 for each organ-related network) samples were used in the following analyses. The soft thresholding measurement method was used, and for protoscoleces obtained from lung CE cysts the *R* ^2^ was 0.58000, which could lead to a loss of information since the WGCNA analyzes are optimized for this type of network. In the case of the liver-related network, it approached the free scale with a *R* ^2^ 0.8350. We found 34 modules in the liver-related network, of which 12 have differential co-expression evidence in lung-related data (Figures 1, 2), namely: DarkGreen, DarkMagenta, DarkRed, LightGreen, MidnightBlue, PaleTurquoise, RoyalBlue, SaddleBrown, SkyBlue, SteelBlue, Violet, and White. These poorly conserved modules between the organ-related networks bring evidence of possible differentially regulated functions in each organ and were used for the following analyses.

**FIGURE 3:**
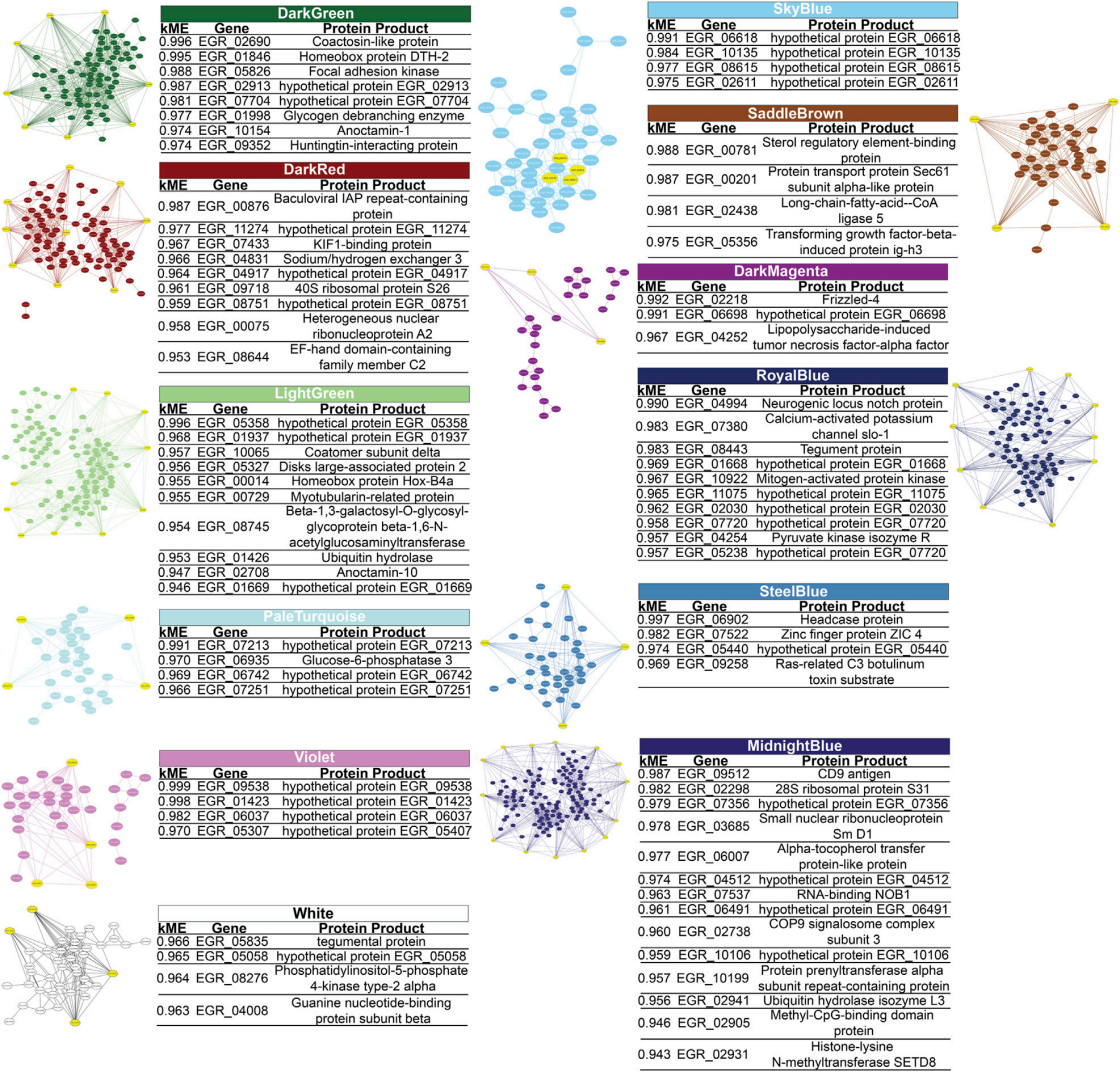
Gene-Gene interaction network of *Echinococcus granulosus* sensu stricto protoscoleces genes from sheep liver CE cysts inside each module. Yellow genes indicate hub genes based on the top 10% genes of each module and high modular membership (kME) values > 0.95. CE, cystic echinococcosis. Three modules contain hub genes related to immune evasion: inside the RoyalBlue module there is the gene that codes for tegument antigen (EGR_08443) and inside the White there is the gene that codes for tegumental protein (EGR_05835), being both genes specific from *E. granulosus* s.l., and inside the MidnightBlue module we found genes for ubiquitin hydrolase isozyme L3 (EGR_02941), COP9 signalosome complex subunit 3 (EGR_02738), tetraspanin CD9 antigen (EGR_09512), and the methyl-CpG-binding protein Mbd2 (EGR_02905).

In each module we determined the hub genes, based on the top 10% genes of each module and high modular membership values (kME > 0.95). Based on this criteria, the DarkGreen module contains 81 genes with 8 hub genes, being all of them known protein products (EGR_02690, EGR_01846, EGR_05826, EGR_02913, EGR_07704, EGR_01998, EGR_10154, EGR_09352), the DarkMagenta module contains 33 genes with 3 hub genes, two of them being known protein products (EGR_02218, EGR_04252) and one hypothetical protein (EGR_06698), the DarkRed module containing 87 genes with 9 hub genes, 6 of them being known protein products (EGR_00876, EGR_07433, EGR_04831, EGR_09718, EGR_00075, EGR_08644) and 3 hypothetical protein (EGR_11274, EGR_04917, EGR_08751), the LightGreen module contains 104 genes with 10 hub genes, being 7 genes with known protein products (EGR_10065, EGR_05327, EGR_00014, EGR_00729, EGR_08745, EGR_01426, EGR_02708) and 3 hypothetical proteins (EGR_05358, EGR_01937, EGR_01669). The MidnightBlue module contains 137 genes with 14 hub genes, 10 of known protein products (EGR_09512, EGR_02298, EGR_03685, EGR_06007, EGR_07537, EGR_02738, EGR_10199, EGR_02941, EGR_02905, EGR_02931) and 4 hypothetical protein products (EGR_07356, EGR_04512, EGR_06491, EGR_10106). The PaleTurquoise module contains 35 genes, with 4 hub genes, being only one (EGR_06935) a known protein product and the rest of them (EGR_07213, EGR_06742, EGR_07251) being hypothetical proteins. The RoyalBlue module contains 89 genes, being 10 of them hub genes, 5 with known protein products (EGR_04994, EGR_07380, EGR_08443, EGR_10922, EGR_04254) and 5 with hypothetical protein products (EGR_01668, EGR_11075, EGR_02030, EGR_07720, EGR_05238). The SaddleBrown module contains 41 genes and 4 hub genes (EGR_00781, EGR_00201, EGR_02438, EGR_05356), no one of these genes were hypothetical proteins. The SkyBlue module shows 43 genes, being 4 of them hub genes (EGR_06618, EGR_10135, EGR_08615, EGR_02611) corresponding all to hypothetical proteins. The SteelBlue module contains 40 genes, with 4 hub genes, all of them corresponding to known protein products (EGR_06902, EGR_07522, EGR_05440, EGR_09258). In the Violet module we found 35 genes, with 4 hub genes being all hypothetical proteins (EGR_09538, EGR_01423, EGR_06037, EGR_05307). Finally, in the White module we found 44 genes, 4 of them are hub genes, 3 of them with known protein products (EGR_05835, EGR_08276, EGR_04008), and only 1 as a hypothetical protein (EGR_05058). These results are summarized in Figure 3.

### Hypothetical protein similar sequences

Analyzing the percentage of hypothetical proteins involved as Hub Genes in each module, two modules (SkyBlue and Violet) contain 100% of these kinds of proteins. PaleTurquoise module shows 75% of Hypothetical proteins as Hub Genes. In the Royal blue, 50% of hub genes belongs to hypothetical proteins. The Midnightblue, DarkMagenta, DarkRed and LightGreen modules presents 36%, 33%, 33% 30% of Hypothetical Proteins as Hub Genes, respectively. Finally, DarkGreen, SteelBlue and White Modules has Hypothetical Proteins as Hub genes in a 25% of the total hub genes. The SaddleBrown module did not show any Hypothetical Protein as Hub gene inside the module.

Since there are many hypothetical proteins as hub genes in the modules, we also performed a sequence similarity search with the Basic Local Alignment Search Toll (BLAST) in each hypothetical protein from each module. In the DarkGreen module EGR_02913 has a 90,91% of identity with *Hymenolepis microstoma* genome assembly, chromosome: 4 (*H*. *microstoma*) and EGR_07704 has 82,3% of identity with *H*. *microstoma* genome assembly, chromosome: 1 (*H*. *microstoma*), both with a Query cover less than 2%. Inside the RoyalBlue module, the EGR_01668 gene has a 79.62% of identity with *Taenia asiatic*a clone TaHC4-G1 mRNA sequence (*T*. *asiatica*) and a Query cover of 63%, and EGR_11075 has an identity of 81.75% with *E*. *granulosus* s.l. SH2 domain-containing protein 4A (EGR_05675), partial mRNA (*E*. *granulosus* s.l.) with a Query cover of 28%. In the SkyBlue module, the EGR_06618 gene has 100% of identity with *Echinococcus multilocularis* DNA, microsatellite EMms2 (*E*. *multilocularis*) and the EGR_10135 gene have 95,54% of identity with *E*. *granulosus* s.l. hypothetical protein (EGR_10285), partial mRNA (*E*. *granulosus* s.l.), both with a Query cover less than 2%. Finally, inside the Violet module the EGR_09538 presents 78,69% of identity with *E*. *granulosus* s.l. hypothetical protein (EGR_05124), partial mRNA (*E*. *granulosus* s.l.) with a Query cover of 100% and the EGR_05307 gene has 26 matches with identity percentage between 80.28% and 100% but a Query cover of less than 18% with other genes from *E*. *granulosus* s.l. No matches were found inside other modules. A detailed view of the gene orthology may be seen in Table 1.

**TABLE 1:** Hypothetical protein gene orthology found in the twelve modules^a^ of protoscoleces from liver CE cysts.

Gene ID	Species	Description	Total score	Query cover	E value	Identity (%)	Accession N^o^.
DarkGreen Module							
EGR_02913	*Hymenolepis microstoma*	*Hymenolepis microstoma* genome assembly, chromosome: 4	87.9	0%	1.00 E−11	90.91	LR215995.1
EGR_07704	*Hymenolepis microstoma*	*Hymenolepis microstoma* genome assembly, chromosome: 1	99	2%	3.00 E−15	82.3	LR215989.1
DarkMagenta Module							
EGR_06698	No results						
DarkRed Module							
EGR_11274	No results						
EGR_04917							
EGR_08751							
LightGreen Module							
EGR_05358	No results						
EGR_01937							
EGR_01669							
PaleTurquoise Module							
EGR_07213	No results						
EGR_06742							
EGR_07251							
RoyalBlue Module							
EGR_01668	*Taenia asiatica*	*Taenia asiatica* clone TaHC4-G1 mRNA sequence	503	63%	1.00 E−137	79.62	EF420605.1
EGR_11075	*Echinococcus granulosus*	*Echinococcus granulosus* SH2 domain-containing protein 4A (EGR_05675), partial mRNA	209	28%	2.00 E−49	81.75	XM_024494924.1
EGR_02030	No results						
EGR_07720							
EGR_05238							
SkyBlue Module							
EGR_06618	*Echinococcus multilocularis*	*Echinococcus multilocularis* DNA, microsatellite EMms2	1759	7%	1.00 E−82	100	XM_024495867.1
EGR_10135	No results						
EGR_08615							
EGR_02611							
SteelBlue Module							
EGR_05440	No results						
Violet Module							
EGR_09538	No results						
EGR_05307	*Echinococcus granulosus*	*Echinococcus granulosus* clone Eg-fos-43 sequence, complete sequence	337	6	3.00 E−87	97.47	KC585049.1
		*Echinococcus granulosus* clone Eg-fos-02 sequence, complete sequence	593	6	9.00 E−78	94.95	KC585045.1
		*E. granulosus* EgBRep repetitive DNA element	305	6	9.00 E−78	94.47	X67152.1
		*E. granulosus* EgDRep repetitive DNA element	303	6	3.00 E−77	94.47	X67153.1
		*Echinococcus granulosus* clone Eg-fos-22 sequence, complete sequence	547	7	5.00 E−75	91.63	KC585042.1
		*Echinococcus granulosus* clone Eg-fos-45 sequence, complete sequence	276	6	7.00 E−69	92.35	KC585050.1
		*Echinococcus granulosus* Peripheral plasma membrane protein CASK (EGR_01323), partial	226	6	7.00 E−54	87.82	XM_024490572.1
		*Echinococcus granulosus* Calmodulin (EGR_01226), partial mRNA	185	4	1.00 E−41	93.60	XM_024490475.1
		*Echinococcus granulosus* Nucleoside diphosphate kinase A 2 (EGR_05582), partial mRNA	183	4	4.00 E−41	93.55	XM_024494831.1
		*Echinococcus granulosus* Neurogenic locus notch protein (EGR_04994), partial mRNA	176	4	7.00 E−39	92.62	XM_024494243.1
		*Echinococcus granulosus* GST (GST) gene, promoter and 5′ untranslated region	147	3	6.00 E−30	90.83	AY174162.1
		*Echinococcus granulosus* DNA-binding protein HEXBP (EGR_00594), partial mRNA	58.4	1	0.003	97.06	XM_024489843.1
		*Echinococcus granulosus* Ecdysone-induced protein 78C (EGR_04406), partial mRNA	56.5	1	0.01	100	XM_024493655.1
		*Echinococcus granulosus* prokaryotic DNA topoisomerase (EGR_03189), partial mRNA	56.5	1	0.01	100	XM_024492438.1
EGR_01423	No results						
EGR_06037							
White Module							
EGR_05058	No results						

^a^ MidnightBlue and SaddleBrown modules had no hypothetical proteins.

## Discussion

Despite morphological and pathophysiological differences in *E*. *granulosus* s.l. protoscoleces from lung and liver that have been previously documented [8, 24, 25], molecular processes associated with this issue has never been addressed based on next-generation sequence data. WGCNA is a method for the analysis of the gene expression patterns of multiple samples, clustering genes and form modules by similar gene expression patterns, creating co-expression networks and identifying intramodular hub genes (highly connected genes inside each module). The main differences among classical differential expression analysis and functional enrichment analysis, is that they cannot reveal connections and interactions among genes that are crucial in biological processes [26, 27]. A recent work involving 10 parasitic platyhelminths (strobilated and non-strobilated) species, identified a set of 34 evolutionary conserved cestode proteins, as possible components of developmental pathways required for strobilation, including *E*. *granulosus* s.l. inside the group of strobilated platyhelminths [28], This work is the only one to explore in this way some aspects of the *E*. *granulosus* s.s. development, but did not assess differences among parasites collected from different viscera. A previous transcriptome work did not identify differential gene expression among viscera in protoscoleces from sheep liver CE cyst and sheep lung CE cyst [23].

From the identified modules of this work, there are two interesting approaches for the discussion. The first one is to look for immune related hub genes in the modules, that can add information about the host-parasite relationship and/or immunoregulation mechanisms. The second one is the hypothetical proteins found as hub genes, as interesting targets for the study of specific and unknown mechanisms of parasitism in each organ.

On the first approach we may highlight the RoyalBlue, White and specially the MidnightBlue modules, in first two modules there is an immune related hub gene specific to *E*. *granulosus s.l*., which is composed of tegument protein and tegumentary antigen. The tegumentary antigen is an immunomodulatory molecule associated with chronic infection, as it inhibits chemotaxis, induces IL-4-positive T lymphocytes and non-complement fixing antibodies (IgG4) [29]. As a specific protein from *E*. *granulosus s.l.*, this data suggest that suppression of a co-expressed gene may consequently also suppress the tegumentary antigen, but it has been demonstrated in our work that it will occur in liver CE cysts and could not happen in lung CE cysts.

In the MidnightBlue module, there are two genes related to protein degradation pathway, first, the ubiquitin hydrolase isozyme L3 (EGR_02941) has been reported before, highlighting that this protein can hydrolyze UBB(+1), (a form of ubiquitin associated with neurogenerative disorders) which is not effectively degraded by the proteasome [30]. On the other hand, we also found the COP9 signalosome complex subunit 3 (EGR_02738), an essential component of COP9 signalosome. This COP9 signalosome is a conserved molecule found in various disease-causing protozoans such as *Leishmania* spp., *Trypanosoma* spp., *Toxoplasma* spp., *and Entamoeba histolytica*. Moreover, in the latter, it was found to be essential for the normal functioning and a druggable parasite target, since disruption of the COP9 from the parasite, leads to a disrregulation of the ubiquitin proteasome pathway, impairing degradation of the proteins and leading to cell death [31]. Also, the COP9 complex has been involved in the immune response, regulating the NF-kB function by protecting the IκBα (an inhibitor of NF-kB) from degradation, leading to reduction of the NF-kB under TNF stimulation [32]. With those two Hub genes inside this module, we speculate that this non-conserved module represents an important part of the protein degradation pathway and could be useful for the parasite to suppress the immune response from the host. Interestingly, NF-κB signaling pathway has particular relevance to several liver diseases and hepatoprotective agents, which could explain why the MidnightBlue module would be specific to this organ [33].

In the same context of the immune response, inside the MidnightBlue module, it is important to note that the tetraspanin CD9 antigen (EGR_09512) acts also as a hub gene in this module. It is known that mononuclear phagocytes fuse to form multinucleated giant cells, a hallmark of the immune response in the adventitial layer of non-fertile CE cysts [34]., When these cells cannot eliminate pathogens, cell-mediated immunity is activated and mononuclear phagocytes coalesce to form Langhans giant cells [35]. A study investigated the role of this tetraspanin associated with CD81 tetraspanin, finding that CD9 and CD81 coordinately prevent the fusion of mononuclear phagocytes [36]. These results suggest a potential role of tetraspanins associations in the immune evasion of the host.

The methyl-CpG-binding protein Mbd2 (EGR_02905) is another important hub gene found in the MidnightBlue, in a study performed in bone marrow-derived dendritic cells (DC) from wild-type (WT) and Mbd2^−/−^ mice, the DC from Mbd2^−/−^ mice presented different mRNA expression, with 70 genes downregulated and 49 genes upregulated (compared with WT). Among these downregulated genes, many of them were related to immunological processes like antigen presentation, showing that this gene is able to induce control over other genes and also suggesting that the inhibition of Mbd2 may impair the optimal DC function and the initiation of a Th2 immune response (which is the main response against helminths [37]). It is curious that a parasite expresses a gene which is responsible of maintaining an adequate immune response against helminthes. We hypothesize that the parasite molecule can mimics host molecule and then evading the immune response, although more studies are needed to confirm this.

With these findings we may think that the MidnightBlue module is important in the immune evasion response and may represent possible therapeutic targets, as all hub genes inside the module are highly connected. This approach opens a window to new therapeutic strategies, as we found specific gene interaction occurring only in protoscoleces from liver but not in lung CE cysts. These differences should be taken into account as the generation of the cyst in the liver may involve different pathways than the occurring in the lung, so it is not misguided to think, as it seems to be a different process of cyst development, as different strategies to control the liver and lung echinococcosis.

Another interesting approach is that SkyBlue and Violet hub genes are exclusively Hypothetical proteins. It means that there are a group of unknown function proteins performing key roles in the parasitism of protoscolex in liver CE cysts, that are not co-expressed in lung CE cysts protoscoleces. This finding also suggests that the microenvironment promoted by the host induces changes in parasite gene expression, which could not be identified by more conventional transcriptomic analyses [23]. As no significative results of homology analysis, we may think that there are many genes of the unknown function that should be explored.

An important limitation of our work that is important to know, is that this approach needs mainly RNA-seq data that considers large number of replicates with low variation among them [28]. Despite this difficulties, choosing a topology close to a scale free-network seems to be a good model to work on, as despite universality of scale-free networks remains controversial, biological networks appear strongly scale free [38]. Conversely, while it is true that would be better to work with more samples, our data will be available for future exploration complemented with more samples obtained from other researchers.

A second approach is to find non-conserved modules in the protoscoleces from lung CE cysts compared to protoscoleces from liver CE cysts. Attempts to perform were unsuccessful, as it was not possible to get a scale-free network in protoscoleces from lung CE cysts, which could lead to a loss of information since the WGCNA analyzes are optimized for this type of network. However, the impossibility of obtaining the scale-free network only prevented an analysis of the conservation of the data of protoscoleces from liver CE cysts data for the identified modules in protoscoleces from lung CE cysts. Thus, data regarding the possible existence of new protoscoleces from lung CE cyst modules, which are not identified in protoscoleces from liver CE cysts, were not included in this work.

Nonetheless, the investigation into hub genes presents a promising avenue for translational research, potentially informing the development of novel therapeutic or control strategies against *E. granulosus*. A pertinent example is the work of Cancela et al. [39], who identified a novel subfamily of nuclear receptors with two DNA-binding domains (2DBDs)—a feature not yet reported in vertebrates. Their elucidation of the full-length 3D structure of the, Eg2DBDα.1 nuclear receptor in *E. granulosus* offers valuable insights into the receptor and structure-function relationship, highlighting its potential as a target for novel anthelmintic drugs.

This research contributes to the understanding of differential gene expression regulation in *E. granulosus*, underscoring the potential role of the organ-specific environment in exerting selective pressure on the parasite. Such environmental influences may have been pivotal in the evolution of certain *E. granulosus* s.l. lineages, enhancing their infective efficacy under specific conditions.

## Conclusion

This work shows that there are differences in gene expression in protoscoleces from Cystic Echinococcosis cysts found in the liver and lungs. We found 34 modules in protoscoleces from liver CE cysts, 12 of which showed differential co-expression compared to protoscoleces from lung CE cysts. Several of these differentially co-expressed modules contain genes related to immune evasion as well as hypothetical proteins of unknown function that are co-expressed exclusively in protoscoleces from liver CE cysts. This suggests there are molecular differences in protoscoleces based on the organ environment of the CE cyst, which has implications for understanding and controlling CE infection.

## Data Availability

The original contributions presented in the study are included in the article/supplementary material, further inquiries can be directed to the corresponding author.
